# Occult Nodal Metastasis in Oral Cavity Cancers

**DOI:** 10.7759/cureus.11640

**Published:** 2020-11-23

**Authors:** Asif Ali Arain, Muhammad Shaheryar Ahmed Rajput, Shakil Akhtar Ansari, Zafar Mahmood, Ahmad Nawaz Ahmad, Muhammad Razzaq Dogar, Anwar Suahil

**Affiliations:** 1 Otolaryngology, The Indus Hospital, Karachi, PAK; 2 Otolaryngology, Liaquat University of Medical and Health Sciences, Jamshoro, PAK; 3 Otolaryngology and Head and Neck Surgery, King Faisal Specialist Hospital and Research Centre, Riyadh, SAU; 4 Otolaryngology and Head and Neck Surgery, Aga Khan University Hospital, Karachi, PAK; 5 Otolaryngology, Liaquat College of Medicine and Dentistry, Darul Sehat Hospital, Karachi, PAK; 6 Otolaryngology, Liaquat National Hospital, Karachi, PAK; 7 Otolaryngology, Jinnah Sindh Medical University, Karachi, PAK

**Keywords:** nodal metastasis, occult regional metastasis, nodal spread, squamous cell neoplasm, squamous cell carcinoma, tongue cancer, cheek cancer, oral cancer, elective neck dissection

## Abstract

Introduction: In squamous cell carcinoma (SCC) of the oral cavity, there is always a risk of occult metastasis to neck nodes in the clinically and radiologically negative neck (N0). Therefore, elective neck dissection (END) has ever been under discussion since the beginning of their routine use for the management of neck for oral carcinomas. The purpose of the current study is to identify the percentage of occult nodal metastasis to neck levels I-V in the cases of oral carcinoma who were treated for the N0 with END.

Methods: Patients who were treated between June 2005 and May 2010 with END from neck levels I to V for the management of N0 with oral SCC had been identified from the database of Aga Khan University Hospital. Those who met the inclusion and exclusion criteria were included in the study. Data were analyzed using SPSS 16 software. Using descriptive statistics, the mean was computed for the quantitative variable (age). Frequencies and percentages were calculated for gender, site, tumor grade, and lymph node involvement for each neck level.

Results: A total of 50 patients were included in the study. There were 38 males and 12 females. The mean age was 47 (range 25-72). The most common site of the tumor was buccal mucosa in 50% of the cases followed by tongue 20%, then floor of mouth 14%, dentoalveolar ridge 8%, retromolar area 4%, lip 2%, and hard palate 2%. Histopathological grading of tumors showed well-differentiated 28%, moderately differentiated 33%, and poorly differentiated 6%. Twenty-seven out of 50 patients were found positive for nodal metastasis on final postoperative histopathology. Neck node metastasis at level I was found in 22 patients, at level II in 16 patients, at level III in seven patients, and at level IV in two patients. The level V was found free of metastasis in all of the cases.

Conclusion: The rate of occult metastatic disease to the neck nodes was similar to that found in the literature. Both early and advanced local disease is associated with a risk of occult metastasis. END for neck levels I-V is, therefore, recommended for the management of the N0 in all cases of oral SCCs. Spread to levels IV and V is rare and these levels should not be a part of routine END.

## Introduction

The oral cavity is the most common site of malignant tumors of the head and neck region [1]. The most common histological type seen in oral cavity malignancies is oral squamous cell carcinoma (OSCC) [2]. These tumors often present with nonhealing indurated lesions in the oral cavity which make the diagnosis relatively easy to suspect but surprisingly the diagnosis of advanced disease at first presentation is not uncommon [3]. Within the diagnosis of OSCC there exist some histological subtypes. Each of the subtypes carries a different prognosis such as verrucous carcinoma which bears a better prognosis whereas the basaloid type is worse [4-5]. Tumor grading into well, moderate, and poorly differentiated carcinomas is of limited prognostic value.

Histopathological factors of prognostic importance include tumor thickness, extra-capsular spread (ECS) of nodal metastasis, and patterns of invasion [6]. In the squamous cell carcinoma (SCC) of the oral tongue, the tumor thickness of more than 4 mm signifies a greater than 20% risk of regional nodal metastasis [7]. Local regional recurrence, distant metastasis, and decreased survival have been consistently reported in association with the ECS in cervical lymph nodes. The pattern of invasion in OSCC should be reported as cohesive, noncohesive, or widely dispersive [8]. The presence of noncohesive invasive front and perineural invasion indicate a high risk of loco-regional relapse [9]. A crude estimate of survival for two years is around 85, 70, 50, and 40% for stage I, II, III, and IV disease respectively [10].

The standard of care is primary surgical resection with or without adjuvant therapy. Improvements in surgical techniques combined with the routine use of postoperative radiation or chemoradiation therapy resulted in improved survival statistics over the last four decades. The challenges involved in the management of such cases require a multidisciplinary approach. A final plan is reached through consensus with the patient and carers. The overall treatment intention, either curative or palliative, needs to be clearly communicated at the beginning. For curative intention, surgical resection should be performed and may be followed by adjuvant therapy if indicated on the histopathological staging of the disease [11].

Even in the cases of early diagnosis with low disease burden (T1 and T2), there remains a risk of metastasis to regional lymph nodes. Occult metastasis had been reported in more than 30% of OSCC with clinically and radiologically negative neck (N0) [12-13]. Therefore, the decision for elective neck dissections (ENDs) for the N0 neck turned out to be difficult and remained under discussion so far. Literature had shown the reduction of survival by as low as 50% in the presence of neck node-positive disease which further underlines the significance of nodal metastasis as the most significant and independent prognostic factor. The purpose of the current study is to identify the percentage of occult nodal metastasis to neck levels I-V in the cases of OSCC who were treated for N0 neck with END.

## Materials and methods

The database of Aga Khan University Hospital was searched to identify patients diagnosed with SCC of the oral cavity who underwent surgical resection between June 2005 and May 2010. The criteria for inclusion and exclusion are shown in Table 1.

**Table 1:** Inclusion and exclusion criteria.

Inclusion criteria	Exclusion criteria
Biopsy proven oral cavity squamous cell carcinoma	Patients who received prior radiation therapy
No radiological or clinical evidence of neck node involvement	Recurrent disease
Patients who underwent elective lymph node dissection to include level I through level V	

Data were analyzed using SPSS 16 software. Using descriptive statistics, the mean and standard deviation were computed for a quantitative variable (age). Frequencies and percentages were calculated for gender, site, tumor grade, and lymph node involvement for each neck level from levels I-V.

## Results

A total of 50 patients were included in the study. There were 38 males and 12 females. The mean age was 47. The minimum age was 25 years, and the maximum age was 72 years. These variables are shown in Table 2. 

**Table 2:** Age and gender distribution.

Variables		Results
Age	Male	38
	Female	12
Gender	Mean	47 years
	Range	25-72 years

The most common site of the tumor was buccal mucosa in 50% of the cases followed by tongue 20% then the floor of mouth 14%, dentoalveolar ridge 8%, retromolar area 4%, lip 2%, and hard palate 2%. This is shown in Table 3.

**Table 3:** Site of tumor.

Site	Number of patients	Percentage
Buccal mucosa	25	50%
Tongue	10	20%
Floor of mouth	7	14%
Dentoalveolar ridge	4	8%
Retromolar area	2	4%
Lip	1	2%
Hard palate	1	2%

Histopathological grading of tumors showed well-differentiated 28%, moderately differentiated 33%, and poorly differentiated 6%, as shown in Table 4.

**Table 4:** Tumor grades.

Grade	Number of patients	Percentage
Well differentiated	14	28%
Moderately differentiated	33	66%
Poorly differentiated	3	6%

Twenty-seven out of 50 patients found positive for nodal metastasis on final postoperative histopathology, as shown in Table 5.

**Table 5:** Post-operative status of neck.

Nodal metastasis	Number of patients	Percentage
Positive	27	54%
Negative	23	46%

Neck node metastasis at level I was found in 22 patients, at level II in 16 patients, at level III in seven patients, and at level IV in two patients. The level V was found free of metastasis in all of the patients. The pattern of nodal metastasis from level I to level V is shown in Table 6. 

**Table 6:** Pattern of nodal metastasis from level I to level V.

Neck levels	Number of patients with metastasis	Percentage
Level I	22	44%
Level II	16	32%
Level III	7	14%
Level IV	2	4%
Level V	0	0%

The majority of our patients had locally advanced disease. The tumor size according to American Joint Committee on Cancer (AJCC), 8th edition, is shown in Table 7 [14].

**Table 7:** Tumor size.

Tumor size	Number of patients	Percentage
T1	5	10%
T2	14	28%
T3	17	34%
T4	14	28%

## Discussion

The critical importance of neck metastasis in OSCC for survival and locoregional control of the disease is a widely demonstrated fact. Therefore, END has always been under discussion since the beginning of its routine use for the management of N0 neck [15-16]. The meta-analysis of four randomized controlled trials evaluating the disease-specific mortality rates in patients with OSCC found significantly reduced death rates in patients who were treated with END for N0 neck [17]. Regional recurrence and poor prognosis had been reported in greater numbers with an observation approach instead of END [18]. Smaller tumors (T1 and T2) are equally aggressive with a reported 30% incidence of nodal metastasis [19]. In particular, the tongue and retromolar area were found to be worse for the possibility of occult metastasis. Byers et al. reported that the tongue was the most common site for occult metastasis [12].

In the current study, 27 out of 50 patients with radiological N0s were found positive on final histopathology after END. The overall percentage of occult metastasis was found to be 54% which is similar to several other studies. Jang et al. reported a 58% incidence of occult metastasis. The authors further correlated the tumor size with occult nodal metastasis and reported significant risk in both early and advanced tumors [20].

There is no method of imaging or other examination technique except histopathology that can detect microscopic foci of metastatic spread in cervical lymph nodes. Immunohistochemical and molecular analysis are superior for detecting occult metastases than light microscopy with ordinary hematoxylin and eosin staining [21-22]. When the probability of occult lymph node metastasis is more than 20%, the guidelines recommend END [23]. However, Okura et al. recommend a probability of more than 44% as a cut-off for END [24]. Either surgery or radiation therapy can be used for the elective treatment of the N0 neck. However, surgery offers a distinct benefit over elective radiation therapy that make surgery the method of preferential choice. Surgery makes the pathological staging of the neck possible which will allow for better decision making regarding the need for adjuvant therapy. If no adjuvant therapy is needed this will improve overall quality of life [21, 25].

Metastasis to levels IV and V cervical lymph nodes is rarely seen. Therefore, neck dissection for these levels is not routinely recommended based upon this study. The recommended procedure of choice for END is a clearance of levels I-III, i.e. supraomohyoid neck dissection. This is also consistent with the findings in our study where none of the patients had metastasis at level V and only 2 had metastasis at level IV [21].

## Conclusions

The findings of the current study revealed that the proportion of patients with occult neck metastasis is considerably high not only in locally advanced cases but also in the early-stage disease. Therefore, elective neck treatment should be considered in all cases of OSCC. Considering the patterns of occult metastasis at each level, the selective neck dissection for levels I-III is recommended for END.
